# C-reactive protein is superior to fecal biomarkers for evaluating colon-wide active inflammation in ulcerative colitis

**DOI:** 10.1038/s41598-021-90558-z

**Published:** 2021-06-14

**Authors:** Natsuki Ishida, Tomohiro Higuchi, Takahiro Miyazu, Satoshi Tamura, Shinya Tani, Mihoko Yamade, Moriya Iwaizumi, Yasushi Hamaya, Satoshi Osawa, Takahisa Furuta, Ken Sugimoto

**Affiliations:** 1 First Department of Medicine, Hamamatsu University School of Medicine, Hamamatsu, Shizuoka Japan; 2 Department of Laboratory Medicine, Hamamatsu University School of Medicine, 1-20-1 Handayama, Higashi-ku, Hamamatsu, Shizuoka 431-3192 Japan; 3 Department of Endoscopic and Photodynamic Medicine, Hamamatsu University School of Medicine, Hamamatsu, Shizuoka Japan; 4 Center for Clinical Research, Hamamatsu University School of Medicine, Hamamatsu, Shizuoka Japan

**Keywords:** Gastroenterology, Inflammatory bowel disease

## Abstract

We evaluated the association between endoscopic scores of colonic inflammation and fecal calprotectin (FC), fecal immunochemical occult blood test (FIT), and C-reactive protein (CRP) in patients with ulcerative colitis (UC). Endoscopic scores reflecting the most severe lesion [maximum Mayo Endoscopic Subscore (M-MES) and Ulcerative Colitis Endoscopic Index of Severity (UCEIS)] and those reflecting the inflammation of the entire colon [sum of MES (S-MES) and Ulcerative Colitis Colonoscopic Index of Severity (UCCIS)] were evaluated. FC, FIT, and CRP were measured, and their association with the four endoscopic scores was evaluated. Endoscopic scores of 78 complete colonoscopies (66 UC patients) were evaluated using the three biomarkers. FC and CRP tended to correlate more strongly with S-MES and UCCIS than with M-MES and UCEIS. In the M-MES 0, 1 group, compared to CRP, FC and FIT showed stronger correlations with S-MES and UCCIS. Conversely, in the M-MES 2, 3 group, only CRP was significantly correlated with each descriptor. CRP more strongly reflects colon-wide mucosal inflammation than FC and allows reliable assessment of inflammation throughout the colon in active UC.

## Introduction

Ulcerative colitis (UC) is an idiopathic chronic inflammatory bowel disease (IBD) with recurrent symptoms of remission and relapse^1^. Since the goal of current treatment for IBD, including UC, is to achieve mucosal healing, it is important to accurately evaluate the inflammatory state of the mucosa in UC^2^. While endoscopy can be used for direct evaluation of the inflammatory state of the intestinal mucosa in UC, biomarkers indirectly reflect the state of inflammation in the colonic mucosa and can be used as a substitute for colonoscopy. C-reactive protein (CRP) is a biomarker that reflects systemic inflammation, which has been evaluated for its usefulness as a marker of IBD activity^3–9^. Recent studies have also described the usefulness of biomarkers in fecal samples, such as fecal calprotectin (FC), and the fecal immunochemical occult blood test (FIT), and reported that these biomarkers more accurately reflect local inflammation of the colon in patients with UC^10–20^.

However, endoscopic assessment is the most accurate way to assess the state of inflammation in UC. Common endoscopic scores for assessing UC activity are the Mayo Endoscopic Subscore (MES) and the Ulcerative Colitis Endoscopic Index of Severity (UCEIS)^21,22^, which represent the scores of the most inflamed lesions in UC. In recent years, new scoring methods for evaluating the inflammatory state of the entire colon have been reported. Kawashima et al. reported the sum of the MES (S-MES), which is the sum of the MES in five colonic segments (ascending, transverse, descending, sigmoid, and rectum), and reported the usefulness of FC in comparison with this new scoring system^23^. Lobatón et al. also evaluated the usefulness of the modified MES (MMES)^24^. Similar to the S-MES, the MMES involves totaling the MES in five segments, multiplying that number by the number of evaluated segments, and dividing the derived number by the number of inflamed segments. The MMES has been clinically, biologically, and histologically correlated with disease activity in UC. The UCEIS is calculated by totaling three descriptors, while the Ulcerative Colitis Colonoscopic Index of Severity (UCCIS) is calculated by scoring four descriptors in five segments and applying the scores in the formula for UCCIS^25,26^. UCCIS is rarely used in clinical practice because of the complexity of scoring. However, scoring is performed by applying the sum of descriptors to the calculation formula, allowing the objective evaluation of the inflammatory state of the entire colon, which is highly useful in clinical research.

Few reports have shown an association between biomarkers and endoscopic scores for evaluating the entire colon, such as the S-MES and UCCIS. In this study, the usefulness of FC, FIT, and CRP as biomarkers for UC was examined using the maximum MES (M-MES) and UCEIS, which show the most severe UC lesions, and the S-MES and UCCIS, which show the degree of inflammation throughout the entire colon. If biomarkers can be confirmed to correlate with colon-wide inflammation in patients with UC, the information may enable the assessment of active disease without the need for colonoscopy, which is associated with an increased risk of perforation in active disease.

## Methods

### Patient population

Patients with UC who were treated at the Hamamatsu University School of Medicine between February 2019 and November 2020 were enrolled in this study. The diagnosis of UC was performed according to established diagnostic criteria, based on clinical, endoscopic, and histological criteria. Patients with IBD who were not diagnosed with UC but were diagnosed with conditions such as indeterminate colitis or unclassified IBD were excluded. UC patients with a history of colorectal surgery were excluded because S-MES and UCCIS require the observation of the total colon. In addition, patients with acute infectious enterocolitis or regular intake of aspirin and/or other nonsteroidal anti-inflammatory drugs were excluded. Seventy-eight patients with UC who met the above criteria were registered.

All procedures performed in studies involving human participants were in accordance with the ethical standards of the institutional and/or national research committee and with the 1964 Helsinki Declaration and its later amendments or comparable ethical standards. Informed consent was obtained from all individual participants included in the study.

### Study design

This was a prospective cross-sectional study. The purpose of this study was to assess the usefulness of FC, FIT, and CRP as biomarkers for UC by correlating these biomarkers with four endoscopic scores. The primary endpoint of this study was the identification of markers that show a significant correlation with each endoscopic score. The secondary endpoint was the correlation between the three biomarkers and each endoscopic score, divided into two groups (M-MES of 0, 1 and M-MES of 2, 3).

### Endoscopic assessment

Patients with UC underwent bowel preparation consisting of the ingestion of a polyethylene glycol-based electrolyte solution prior to colonoscopy. UC endoscopic scores were assessed using the M-MES, S-MES, UCIES, and UCCIS. M-MES was graded as follows: 0, normal or inactive disease; 1, mild disease with erythema, decreased vascular pattern, and mild friability; 2, moderate disease with marked erythema, absence of vascular patterns, friability, and erosions; and 3, severe disease with spontaneous bleeding and ulceration in the lesion with the most severe inflammation^21^. S-MES was calculated by totaling the MES in five colonic segments (ascending, transverse, descending, sigmoid, and rectum), as described above^23^.

The UCEIS score was calculated as the sum of three descriptors: vascular pattern (score 0–2), erosions and ulcers (score 0–3), and bleeding (score 0–3)^22^. The UCCIS score was assessed using the following descriptors in the fives segments, as in the S-MES: vascular pattern (score 0–2), granularity (score 0–2), erosions and ulcers (score 0–4), and bleeding/friability (score 0–2). These descriptor scores were then applied to the following formula: UCCIS = 3.1 × Sum (vascular pattern across five segments) + 3.6 × Sum (granularity across five segments) + 3.5 × Sum (ulceration across five segments) + 2.5 × Sum (bleeding/friability across five segments)^25,26^. M-MES 0 or 1 was defined as indicative of mucosal healing.

### Clinical activity assessment

Clinical disease activity was evaluated using Lichtiger’s clinical activity index (CAI)^27^. CAI was evaluated using the following criteria: the presence of diarrhea (number of stools per day), nocturnal diarrhea, visible blood in stool (percentage of movements), fecal incontinence, abdominal pain or cramping, general well-being, abdominal tenderness, and a need for anti-diarrheal drugs^27^. Clinical remission was defined as a CAI ≤ 3.

### FC measurement

Fecal samples were prepared on or before the day of colonoscopic preparation. Samples were collected in plastic tubes for FC measurement and shipped at − 20 °C, as recommended by the laboratory (SRL, Inc., Tokyo, Japan). FC was measured with a Phadia 250 immunoanalyzer (HITACHI Ltd., Tokyo, Japan) using the Elia A Calprotectin 2 reagent (Phadia GmbH, Freiburg, Germany), via fluorescence enzyme immunoassay principles.

### FIT measurement

To prevent bleeding due to the endoscopic examination, fecal samples were obtained on or before the day of colonoscopic preparation. A collection kit (Eiken Chemical, Tokyo, Japan) was used to collect stool specimens. Submitted samples were immediately processed and examined using the OC Sensor io (Eiken Chemical).

### CRP measurement

According to routine clinical practice, serum CRP level was measured to assess the UC activity status of patients. Blood samples were collected within a few days of endoscopic examination. This measurement was performed at the Laboratory Test Department of Hamamatsu University School of Medicine.

### Statistical analysis

Statistical analyses of the data were performed using SPSS version 24 (IBM Armonk, New York, NY), SAS version 9.4 (SAS Institute, Cary, NC), and EZR (Saitama Medical Center, Jichi Medical University, Saitama, Japan) software. Correlation analyses were performed using Spearman’s rank correlation test. *P* < 0.05 was considered statistically significant.

### Ethical approval

The study protocol was reviewed and approved by the ethics committee of Hamamatsu University School of Medicine (20-322). Further, the investigation was conducted in accordance with Good Clinical Practice principles and in adherence to the Declaration of Helsinki. All patients provided informed consent prior to enrollment in this study.

## Results

### Patient characteristics

Seventy-eight colonoscopies (78 fecal/blood samples) were performed in patients with UC. The baseline characteristics of the patients are shown in Table 1.

**Table 1:** Patient characteristics.

Characteristics	N = 78
Age (year), mean (range) ± SD	47.3 (18‒77) ± 16.7
Male/female, n (%)	47 / 31 (60.3 / 39.7)
Disease duration (year), mean (range) ± SD	10.9 (0.3‒38) ± 10.3
**Disease extent, n (%)**	
Extensive colitis	43 (55.1)
Left-sided colitis	28 (35.9)
Proctitis	7 (9.0)
CAI (Lichtiger’s score) mean (range) ± SD	2.21 (0‒15) ± 2.87
M-MES mean (range) ± SD	0.87 (0‒3) ± 0.86
S-MES mean (range) ± SD	1.86 (0‒8) ± 2.13
UCEIS mean (range) ± SD	1.91 (0‒7) ± 1.94
UCCIS mean (range) ± SD	16.14 (0‒81.2) ± 19.05
FC (µg/g) mean (range) ± SD	3448.3 (8.9‒49,300) ± 7945.5
FIT (ng/mL) mean (range) ± SD	2651.6 (30‒45,900) ± 7195.5
CRP (mg/dL) mean (range) ± SD	0.39 (0.02‒7.23) ± 1.10
**Medication at study, n (%)**	
Oral 5‒ASA	53 (67.9)
Suppository 5‒ASA	9 (11.5)
Systemic steroids	9 (11.5)
Immunomodulators	24 (30.8)
Biologics	25 (32.1)

*CAI* Clinical activity index, *S-MES* Sum of Mayo endoscopic subscore, *M-MES* Maximum Mayo endoscopic subscore, *UCEIS* Ulcerative colitis endoscopic index of severity, *UCCIS* Ulcerative colitis colonoscopic index of severity, *FC* Fecal calprotectin, *FIT* Fecal immunochemical occult blood test, *CRP* C-reactive protein, *5-ASA* 5-aminosalicylic acid.

### Correlations between the biomarkers and endoscopic scores

Correlations between each of the three biomarkers and each of the four endoscopic scores were analyzed (Table 2). Every endoscopic score showed a significant correlation with all biomarkers. In particular, compared to CRP, FC and FIT showed a stronger correlation with each endoscopic score. FC and CRP levels tended to correlate more strongly with S-MES and UCCIS, which represent the inflammation of the entire colon, than with the M-MES and UCEIS, which reflect the most severe colonic lesions in UC (Table 2). In contrast, FIT tended to correlate more strongly with M-MES and UCEIS (Table 2).

**Table 2:** Correlations between biomarkers and endoscopic scores.

	FC		FIT		CRP	
	*r*	*P*	*r*	*P*	*r*	*P*
M-MES	0.694	< 0.001	0.773	< 0.001	0.453	< 0.001
S-MES	0.737	< 0.001	0.767	< 0.001	0.544	< 0.001
UCEIS	0.706	< 0.001	0.813	< 0.001	0.462	< 0.001
UCCIS	0.745	< 0.001	0.749	< 0.001	0.562	< 0.001

*FC* Fecal calprotectin, *FIT* Fecal immunochemical occult blood test, *CRP* C-reactive protein, *r* Correlation coefficient, *S-MES* Sum of Mayo endoscopic subscore, *M-MES* Maximum Mayo endoscopic subscore, *UCEIS* Ulcerative colitis endoscopic index of severity, *UCCIS* Ulcerative colitis colonoscopic index of severity.

### Correlations between the biomarkers and the descriptors of UCIES/UCCIS

To find the factors that were responsible for the correlations between the biomarkers and endoscopic scores (Table 2), we evaluated the correlations between the evaluation items of UCEIS and UCCIS and the three biomarkers (Table 3). For UCCIS, the correlation between the sum of each descriptor in the five colonic segments and each biomarker was examined. Each biomarker showed a significant correlation with all descriptors (Table 3). FIT, which reflects bleeding associated with intestinal inflammation, showed a stronger correlation with bleeding items than FC and CRP.

**Table 3:** Correlations between biomarkers and the descriptors of UCIES/UCCIS.

	FC		FIT		CRP	
	*r*	*P*	*r*	*P*	*r*	*P*
**UCEIS**						
V	0.659	< 0.001	0.723	< 0.001	0.430	< 0.001
E	0.636	< 0.001	0.712	< 0.001	0.453	< 0.001
B	0.514	< 0.001	0.718	< 0.001	0.360	0.001
**UCCIS**						
Sum of V	0.716	< 0.001	0.727	< 0.001	0.494	< 0.001
Sum of G	0.718	< 0.001	0.763	< 0.001	0.511	< 0.001
Sum of E	0.710	< 0.001	0.708	< 0.001	0.532	< 0.001
Sum of B	0.381	< 0.001	0.660	< 0.001	0.308	0.006

*UCEIS* Ulcerative colitis endoscopic index of severity, *UCCIS* Ulcerative colitis colonoscopic index of severity, *FC* Fecal calprotectin, *FIT* Fecal immunochemical occult blood test, *CRP* C-reactive protein, *r* Correlation coefficient, *V* Vascular pattern, *E* Erosions and ulcers, *B* Bleeding, *Sum of V* Sum of vascular pattern, *Sum of G* Sum of granularity, *Sum of E* Sum of erosions and ulcers, *Sum of B* Sum of bleeding/friability.

### Correlation between the biomarkers and S-MES/UCCIS in the M-MES 0, 1 and M-MES 2, 3 groups

We divided all cases into a group with M-MES 0, 1, which is generally considered to reflect mucosal healing in UC, and a group with M-MES 2, 3, which is associated with endoscopic UC activity. In these two groups, the correlations of S-MES and UCCIS with each biomarker were analyzed (Table 4). In the M-MES 0, 1 group, all biomarkers showed a significant correlation with S-MES and UCCIS, with FC and FIT showing a stronger correlation than CRP. Conversely, in the M-MES 2, 3 group, only CRP showed a significant correlation with S-MES and UCCIS.

**Table 4:** Correlations between biomarkers and endoscopic scores divided into two groups—M-MES 0, 1 and M-MES 2, 3.

	FC		FIT		CRP	
	*r*	*P*	*r*	*P*	*r*	*P*
**M-MES 0, 1 (n = 55)**						
S-MES	0.593	< 0.001	0.581	< 0.001	0.292	0.030
UCCIS	0.653	< 0.001	0.672	< 0.001	0.269	0.047
**M-MES 2, 3 (n = 23)**						
S-MES	0.285	0.198	− 0.074	0.744	0.629	0.002
UCCIS	0.259	0.244	0.056	0.805	0.747	< 0.001

*M-MES* Maximum Mayo endoscopic subscore, *FC* Fecal calprotectin, *FIT* Fecal immunochemical occult blood test, *CRP* C-reactive protein, *r* Correlation coefficient, *S-MES* Sum of Mayo endoscopic subscore, *UCCIS* Ulcerative colitis colonoscopic index of severity.

### Correlation between the biomarkers and descriptors of UCCIS in the M-MES 0, 1 and M-MES 2, 3 groups

The correlations between the sum of the descriptors in UCCIS and the three biomarkers were evaluated in the M-MES 0,1 group (Table 5). FC and FIT showed significant correlations with each item, except for the sum of bleeding/friability. CRP did not show a significant correlation with any of the UCCIS items. Similarly, the correlations between the sum of the descriptors in UCCIS and the three biomarkers were evaluated in the M-MES 2, 3 group (Table 6). FC and FIT did not show a significant correlation with the sum of the UCCIS descriptors. CRP showed a significant correlation with all descriptors, except for the sum of bleeding/friability. In patients with active UC, CRP did not reflect intestinal bleeding but strongly reflected other inflammatory conditions of the colon.

**Table 5:** Correlations between biomarkers and the descriptors of UCCIS in the MES 0, 1 group.

	FC		FIT		CRP	
	*r*	*P*	*r*	*P*	*r*	*P*
**UCCIS**						
Sum of V	0.699	< 0.001	0.568	< 0.001	0.252	0.063
Sum of G	0.611	< 0.001	0.586	< 0.001	0.257	0.058
Sum of E	0.594	< 0.001	0.608	< 0.001	0.243	0.074
Sum of B	0.051	0.709	0.232	0.088	− 0.004	0.975

*UCCIS* Ulcerative colitis colonoscopic index of severity, *FC* Fecal calprotectin, *FIT* Fecal immunochemical occult blood test, *CRP C-reactive protein*, *r* Correlation coefficient, *V* Vascular pattern, *G* Granularity, *E* Erosions and ulcers, *B* Bleeding, *Sum of V* Sum of vascular pattern, *Sum of G* Sum of granularity, *Sum of E* Sum of erosions and ulcers, *Sum of B* Sum of bleeding/friability.

**Table 6:** Correlations between biomarkers and the descriptors of UCCIS in the MES 2, 3 group.

	FC		FIT		CRP	
	*r*	*P*	*r*	*P*	*r*	*P*
**UCCIS**						
Sum of V	0.042	0.850	− 0.054	0.806	0.742	< 0.001
Sum of G	0.124	0.573	0.118	0.591	0.591	0.003
Sum of E	0.118	0.591	− 0.086	0.698	0.733	< 0.001
Sum of B	− 0.279	0.198	0.446	0.033	− 0.002	0.992

*UCCIS* Ulcerative colitis colonoscopic index of severity, *FC* Fecal calprotectin, *FIT* Fecal immunochemical occult blood test, *CRP* C-reactive protein, *r* Correlation coefficient, *V* Vascular pattern, *G* Granularity, *E* Erosions and ulcers, *B* Bleeding, *Sum of V* Sum of vascular pattern, *Sum of G* Sum of granularity, *Sum of E* Sum of erosions and ulcers, *Sum of B* Sum of bleeding/friability.

## Discussion

In this study, we analyzed the usefulness of FC, FIT, and CRP as biomarkers for UC diagnosis using scores representing the most severe lesion (M-MES and UCEIS) and scores representing inflammation throughout the colon (S-MES and UCCIS). FC and CRP tended to correlate more strongly with S-MES and UCCIS than with M-MES and UCEIS. In the M-MES 0, 1 group, compared to CRP, FC and FIT showed stronger correlations with S-MES and UCCIS. Conversely, in the M-MES 2, 3 group, only CRP correlated significantly with each descriptor.

To date, several endoscopic scores of UC have been reported, but the MES is the most widely used score in clinical practice and trials. In addition, the UCEIS is often used as an endoscopic score for UC. However, these scores present the lesion with the most severe inflammation and do not indicate inflammation in the entire colon^21,22^. There are reports of endoscopic scores that evaluate inflammation throughout the colon. In many of these reports, the colon was divided into five to seven segments, the state of the mucosa was evaluated in each segment, and the score of the entire colon was obtained^18,20,23–26^. However, such scoring systems for evaluating the entire colon are rarely used in clinical practice due to the complexity of evaluation. Endoscopic scores, such as the MES and UCEIS, which are widely used in clinical practice, indicate scores in the areas with the most severe inflammation, whereas biomarkers for UC, which have been reported to be useful in recent years, are considered to reflect inflammation throughout the colon. It is considered important to compare inflammation throughout the colon with biomarkers to evaluate the characteristics of UC biomarkers more accurately.

The four endoscopic scores showed a strong significant correlation with the biomarkers, particularly with FC and FIT. Although CRP showed a less strong correlation than FC and FIT, it showed a significant correlation with the four endoscopic scores. FC and CRP showed a stronger correlation with S-MES and UCCIS, which represent the presence of inflammation in the entire colon, than with M-MES and UCEIS, which indicate the most severe lesion. Kawashima et al. reported that the correlation coefficients between FC and M-MES or S-MES were r = 0.79 and r = 0.86, respectively, indicating that FC may more strongly reflect inflammation throughout the colon^23^. In the study by Lobatón et al., the correlation coefficients of FC with MMES, which is synonymous with S-MES, and M-MES, were *r* = 0.669 and *r* = 0.725, respectively^24^. The correlations between various markers and cumulative MES (cMES), which is calculated by totaling the MESs of six segments (cecum, ascending colon, transverse colon, descending colon, sigmoid colon, and rectum), were analyzed in a previous study^20^. In that study, the correlation coefficients of FC with M-MES and cMES were *r* = 0.38 and *r* = 0.52, respectively, which are similar to our results.

Few studies have evaluated the relationship between the entire colon and various biomarkers. In the present study, FIT tended to have a larger coefficient of correlation with M-MES and UCEIS than with S-MES and UCCIS. Although the cause of these findings is unknown, it may be related to the ability of FIT to reflect the most severely inflamed lesion. Further case accumulation and analysis are therefore required. Furthermore, to examine the factors that reflect the inflammation of each marker, the descriptors of UCEIS and UCCIS were compared with each biomarker. The results of this analysis showed that FIT was strongly correlated with bleeding in the UCEIS and the sum of bleeding/friability in the UCCIS. Naganuma et al. reported similar results to those of the present study^28^. In their study, compared to FC, FIT was more strongly correlated with bleeding in the UCEIS and reflected the association of bleeding with inflammation in UC.

Next, we examined whether the characteristics of biomarkers differ, depending on the presence or absence of endoscopic activity, using widely used endoscopic scores. In this study, mucosal healing was defined as M-MES 0, 1, and the correlations between endoscopic scores and biomarkers were evaluated. In the M-MES 0, 1 group, although FC, FIT, and CRP showed significant correlations with S-MES and UCCIS, the coefficient of correlation of CRP was lower than those of FC and FIT. Notably, in the M-MES 2, 3 group, only CRP showed a significant correlation with S-MES and UCCIS. In other words, from the viewpoint of the use of biomarkers for the evaluation of the inflammation of the entire colon, although it is desirable to use FC or FIT in the mucosal healing phase, evaluation using CRP may be more useful than using other biomarkers in the endoscopic active phase. Similar findings were also shown in a study by Sonoyama et al. that compared FC and CRP with endoscopic scores^29^. In their report, although there was a significant difference in FC values between groups with MES 0 versus MES 1 versus MES 2 in UC patients with extensive colitis, no significant difference was observed between the MES 2 and MES 3 groups. Contrarily, although no significant difference was shown in CRP value between the MES 0 and MES 1 groups, a significant difference was shown in the MES 1 versus MES 2 versus MES 3 groups. The difference between their study and ours is that they did not evaluate the endoscopic score of the entire colon; however, consistent with our findings, the authors found that CRP was more useful in the endoscopic active phase. In addition, as shown in supplemental Table 1, the values of FC, FIT, and CRP were significantly higher in the MES 2, 3 group than in the MES 0, 1 group. Furthermore, although there were significant differences in all biomarkers between the mucosal healing phase and the active phase, the correlation coefficient of CRP was higher than those of FC and FIT, as shown in Table 4, which confirms the lower diagnostic accuracies of FC and FIT compared to that of CRP in the active phase.

Furthermore, to investigate the characteristics of the biomarkers in both the MES 0, 1, and MES 2, 3 groups, the correlation between the sum of descriptors in the UCCIS and each biomarker was analyzed. As expected, FC and FIT showed significant correlations with almost all items in the MES 0, 1 group, whereas these biomarkers did not show correlation with nearly all the descriptors in the MES 2, 3 groups. Conversely, CRP did not show significant correlations with each sum of descriptors in the MES 0, 1 group, whereas a significant correlation of each sum of descriptors was found in the MES 2, 3 group in all items, except for the sum of bleeding/friability. The results of this sub-group analysis reveal that CRP does not reflect the amount of bleeding in the endoscopic active phase but strongly reflects the presence of other factors.

This study had several limitations. First, the sample size was small. In addition, the number of patients with active UC was small. In this study, the entire colon was evaluated in order to sum the MES and UCCIS. However, in clinical practice, the high severity of UC limits the number of cases in which total colonoscopy can be performed, due to the risks of patient distress and complications, such as perforation and bleeding. For this reason, the number of active UC cases was small in this study. However, even with the small number of cases, the significant correlation of CRP with the S-MES and UCCIS in UC patients in the endoscopic active phase indicates that CRP is more strongly correlated with inflammation throughout the colon.

Patients with IBD have a higher risk of perforation than healthy individuals, and colonoscopy, particularly in patients with severe IBD, has the potential of further increasing the risk of perforation^30,31^. Therefore, we considered that CRP is an adequate marker of active colon inflammation, reducing the requirement of colonoscopy, with the associated risk.

In conclusion, CRP more strongly reflects inflammation throughout the colon than FC and FIT in UC patients with severe endoscopic activity. CRP is useful in situations where endoscopy cannot be performed (for example, when gastrointestinal perforation is suspected or during a pandemic, such as in the current COVID-19 pandemic).

## Supplementary information

Supplementary Information.

## Data Availability

Data available upon request from Ken Sugimoto (sugimken@hama-med.ac.jp).

## References

[1] Podolsky DK (2002). Inflammatory bowel disease. N. Engl. J. Med..

[2] Colombel JF (2011). Early mucosal healing with infliximab is associated with improved long-term clinical outcomes in ulcerative colitis. Gastroenterology.

[3] Solem CA (2005). Correlation of C-reactive protein with clinical, endoscopic, histologic, and radiographic activity in inflammatory bowel disease. Inflam. Bowel Dis..

[4] Zilberman L (2006). Correlated expression of high-sensitivity C-reactive protein in relation to disease activity in inflammatory bowel disease: lack of differences between Crohn's disease and ulcerative colitis. Digestion.

[5] Henriksen M (2008). C-reactive protein: a predictive factor and marker of inflammation in inflammatory bowel disease. Results from a prospective population-based study. Gut.

[6] Lok KH (2008). Correlation of serum biomarkers with clinical severity and mucosal inflammation in Chinese ulcerative colitis patients. J. Dig. Dis..

[7] Osada T (2008). Correlations among total colonoscopic findings, clinical symptoms, and laboratory markers in ulcerative colitis. J. Gastroenterol. Hepatol..

[8] Karoui S (2011). Correlation of C-reactive protein with clinical and endoscopic activity in patients with ulcerative colitis. Dig. Dis. Sci..

[9] Uchihara M (2017). Blood biomarkers reflect integration of severity and extent of endoscopic inflammation in ulcerative colitis. JGH Open.

[10] Schoepfer AM (2009). Ulcerative colitis: correlation of the Rachmilewitz endoscopic activity index with fecal calprotectin, clinical activity, C-reactive protein, and blood leukocytes. Inflam. Bowel Dis..

[11] D'Haens G (2012). Fecal calprotectin is a surrogate marker for endoscopic lesions in inflammatory bowel disease. Inflam. Bowel Dis..

[12] Nakarai A (2013). Evaluation of mucosal healing of ulcerative colitis by a quantitative fecal immunochemical test. Am. J. Gastroenterol..

[13] Schoepfer AM (2013). Fecal calprotectin more accurately reflects endoscopic activity of ulcerative colitis than the Lichtiger Index, C-reactive protein, platelets, hemoglobin, and blood leukocytes. Inflam. Bowel Dis..

[14] Mooiweer E, Fidder HH, Siersema PD, Laheij RJ, Oldenburg B (2014). Fecal hemoglobin and calprotectin are equally effective in identifying patients with inflammatory bowel disease with active endoscopic inflammation. Inflam. Bowel Dis..

[15] Takashima S (2015). Evaluation of mucosal healing in ulcerative colitis by fecal calprotectin vs. fecal immunochemical test. Am. J. Gastroenterol..

[16] Ryu DG (2016). Assessment of disease activity by fecal immunochemical test in ulcerative colitis. World J. Gastroenterol..

[17] Patel A, Panchal H, Dubinsky MC (2017). Fecal calprotectin levels predict histological healing in ulcerative colitis. Inflam. Bowel Dis..

[18] Shi HY (2017). Accuracy of faecal immunochemical test to predict endoscopic and histological healing in ulcerative colitis: a prospective study based on validated histological scores. J. Crohns Colitis.

[19] Hiraoka S (2018). Fecal immunochemical test and fecal calprotectin results show different profiles in disease monitoring for ulcerative colitis. Gut Liver.

[20] Mak WY (2018). Fecal calprotectin in assessing endoscopic and histological remission in patients with ulcerative colitis. Dig. Dis. Sci..

[21] Schroeder KW, Tremaine WJ, Ilstrup DM (1987). Coated oral 5-aminosalicylic acid therapy for mildly to moderately active ulcerative colitis. A randomized study. N. Engl. J. Med..

[22] D'Haens G (2007). A review of activity indices and efficacy end points for clinical trials of medical therapy in adults with ulcerative colitis. Gastroenterology.

[23] Kawashima K (2016). Fecal calprotectin level correlated with both endoscopic severity and disease extent in ulcerative colitis. BMC Gastroenterol..

[24] Lobatón T (2015). The Modified Mayo Endoscopic Score (MMES): a new index for the assessment of extension and severity of endoscopic activity in ulcerative colitis patients. J. Crohns Colitis.

[25] Neumann H, Neurath MF (2012). Ulcerative colitis: UCCIS—A reproducible tool to assess mucosal healing. Nat. Rev. Gastroenterol. Hepatol..

[26] Samuel S (2013). Validation of the ulcerative colitis colonoscopic index of severity and its correlation with disease activity measures. Clin. Gastroenterol. Hepatol..

[27] Lichtiger S, Present DH (1990). Preliminary report: cyclosporin in treatment of severe active ulcerative colitis. Lancet.

[28] Naganuma M (2020). Significance of conducting 2 types of fecal tests in patients with ulcerative colitis. Clin. Gastroenterol. Hepatol..

[29] Sonoyama H (2019). Capabilities of fecal calprotectin and blood biomarkers as surrogate endoscopic markers according to ulcerative colitis disease type. J. Clin. Biochem. Nutr..

[30] Buisson A (2013). Colonoscopic perforations in inflammatory bowel disease: a retrospective study in a French referral centre. Dig. Liver Dis..

[31] Navaneethan U (2012). Severe disease on endoscopy and steroid use increase the risk for bowel perforation during colonoscopy in inflammatory bowel disease patients. J. Crohns Colitis.

