# Supplementary material for: C-reactive protein is superior to fecal biomarkers for evaluating colon-wide active inflammation in ulcerative colitis
Source: Sci Rep. 2021 Jun 14;11:12431. doi: 10.1038/s41598-021-90558-z (PMC8203605; doi:10.1038/s41598-021-90558-z)
Supplement: Supplementary file 1 — Supplementary Information. [file 41598_2021_90558_MOESM1_ESM.docx]

Supplemental table 1 Comparison of each biomarker between the M-MES 0, 1 and M-MES 2, 3 groups

|  | M-MES 0, 1 (n = 55) | M-MES 2, 3 (n = 23) | P-value |
| --- | --- | --- | --- |
| FC | 1321.83 ± 2688.6 | 8533.17 ± 12840.9 | <0.001 |
| FIT | 658.5 ± 2424.3 | 7417.8 ± 11537.1 | <0.001 |
| CRP | 0.16 ± 0.31 | 0.96 ± 1.87 | 0.003 |

M-MES, Maximum Mayo Endoscopic Subscore; FC, Fecal calprotectin; FIT, Fecal immunochemical occult blood test; CRP, C-reactive protein.
